# T Cell Subsets and the Expression of Related MicroRNAs in Patients with Recurrent Early Pregnancy Loss

**DOI:** 10.1155/2023/8215567

**Published:** 2023-03-29

**Authors:** Ya-ni Yan, Jian Zhang, Na Yang, Chaochao Chen, Weiwei Li

**Affiliations:** ^1^Department of Reproductive Medicine, Qinhuangdao Maternal and Child Health Care Hospital, Qinhuangdao, Hebei 066000, China; ^2^Jiulongshan Hospital of Qinhuangdao, Qinhuangdao, Hebei 066000, China

## Abstract

This study explored the role of T cell subsets and the expression of related microRNAs in patients with recurrent early pregnancy loss (EPL). Fifty patients with EPL loss between May 2018 and May 2021 were randomly selected as the EPL group, and 50 pregnant women with normal pregnancies or normal delivery outcomes were randomly selected as the control group. The expression levels of T cell subset-related markers and T cell subset-related miRNAs, in addition to the frequencies of T cell subsets, in peripheral blood of the two groups were analyzed. In terms of T cell-related markers, the results showed that the expression levels of the transcriptional regulator TBX-21 (T-bet) and interferon regulatory factor 4 (IRF4) were significantly upregulated in peripheral blood of the patients in the EPL group (*P* < 0.05), whereas the expression levels of GATA binding protein 3 (GATA3) and glucocorticoid-induced tumor necrosis factor receptor (GITR) were significantly downregulated (*P* < 0.05). In the EPL group, the expression of mir-106b, mir-93, and mir-25 was upregulated (1.51 ± 0.129, 1.43 ± 0.132, and 1.73 ± 0.156, respectively) in regulatory T (Treg) cell-related T cell subsets, whereas the expression of miR-146a and miR-155 was downregulated (*P* < 0.05). The frequencies of Treg and exhausted T cells in the EPL group were significantly lower than those in the control group (*P* < 0.05). The cell frequencies of T helper 17 (Th17) cells and exhausted Treg cells in the EPL group were significantly higher than those in the control group (*P* < 0.05). In conclusion, immune cells and associated miRNA profiles can be used as prognostic biomarkers for the treatment of human reproductive disorders, such as EPL.

## 1. Introduction

Early pregnancy loss (EPL) is defined as pregnancy loss within 12 weeks of gestation and includes miscarriages and biochemical pregnancy loss [1]. EPL, which includes spontaneous miscarriages, the most common type of EPL, embryonic abortion, and intrauterine fetal death, accounts for approximately 80% of pregnancy losses [2]. Recurrent EPL is a major concern in human reproduction and the focus of much research due to its complex etiology, poor prognosis, and adverse effects on the physical and mental health of pregnant women [3]. Immune abnormalities, mainly T cell subsets and related miRNA abnormalities, are known to play a vital role in the occurrence of EPL [4, 5].

Studies have shown that regulatory T (Treg) cells play an important role in the process of immune regulation and the survival of the fetus in the uterus [6, 7]. T helper 17 (Th17) cells, a newly recognized subpopulation of effector helper T cells, have the ability to promote host defense against extracellular pathogens and modulate inflammatory, chronic, and autoimmune diseases [6, 7]. A recent study showed that Th17 and Treg cells are strongly associated not only with successful pregnancies but also with pregnancy disorders, such as preeclampsia (PE) and recurrent spontaneous abortion (RSA), in which the frequency of Treg cells is decreased and the frequency of Th17 cells is increased [8]. Hadfield et al. and McCracken et al. showed that in the third trimester of pregnancy, the inability to mount a sufficient Th1 response resulted in diminished expression of the Th1 transcriptional regulator TBX-21 (T-bet) [9, 10]. They also showed that reconstitution of T-bet expression restored cytokine synthesis in T cells.

Some studies in recent years have shown that compared with normal pregnancies, miRNAs in exosomes and plasma of patients with RSA show significant changes [11, 12], in which miR-559, miR-146b-5p, miR-320b, and miR-221-3p were significantly upregulated, while miR-101-3p was significantly downregulated. This finding indicates that miRNAs can regulate RSA through a variety of potential mechanisms involving different target genes and binding sites. However, the specific mechanism is unclear and needs to be further explored. Liu et al. found that the expression of miR-93 and target B cell lymphoma-2-like 2 (BCL2L2) was increased in chorionic villi of RSA patients compared to that in women with normal pregnancies and that BCL2L2 expression adversely affected the balance of apoptosis, cell proliferation, migration, and invasion, promoting the development of RSA [13]. Other research demonstrated that miR-155-5p regulated RSA by activating the nuclear factor kappa-light-chain-enhancer of activated B cells signaling pathway [14]. And the overexpression of miR-155-5p was followed by a decrease in the release of inflammatory cytokines, including interleukin- (IL-) 6, interferon-*γ* (IFN-*γ*), tumor necrosis factor-*α*, and IL-10 from decidual stromal cells, thereby inhibiting the apoptosis of decidual stromal cells [14].

In this study, we explored the role of T cell subsets in the pathogenesis of RPL by evaluating the number of T cells, associated transcription factors, and miRNAs targeting these transcription factors. The results provide data that may aid the treatment and prevention of RPL.

## 2. Materials and Methods

### 2.1. Ethic Approval

The participants were informed about the study and voluntarily agreed to take part. This study complies with the ethical requirements of the Declaration of Helsinki and was approved by the Medical Ethics Committee of Qinhuangdao Maternal and Child Health Care Hospital. Written consent was obtained before conducting the study.

### 2.2. Inclusion Criteria

Pregnant women aged 18–41 years with a clinical diagnosis of recurrent EPL and no abnormalities in spousal semen quality testing were enrolled in the study. Recurrent EPL referred to two or more abortions before the 12th week of pregnancy [15].

### 2.3. Exclusion Criteria

Pregnant women with severe chromosomal abnormalities, acquired immune deficiency syndrome, hepatitis C, hepatitis B, and chronic diseases were excluded, in addition to those with a history of long-term continuous medication control, asthma, drug allergies, cervical dysfunction and uterine malformations, smoking and alcohol abuse, tumors, and other malignant diseases. Those younger than 18 years or older than 41 years were also excluded.

### 2.4. General Data

Fifty patients with recurrent EPL who presented to our hospital between May 2018 and May 2021 and met the inclusion criteria were randomly selected as the EPL group, and another 50 pregnant women with normal pregnancies or normal deliveries were randomly selected as the control group. There was no significant difference (*P* > 0.05) between the two groups in terms of age, nationality, parity, body mass index, or other general data (Table 1).

### 2.5. Assessment and Detection of Natural Killer (NK) Cell Expression Frequency

The cytotoxicity of NK cells was assessed by flow cytometry using K562 target cells. First, peripheral blood mononuclear cells (PBMCs) were isolated and incubated with prestained K562 target cells and propidium iodide at 37°C and 5% CO_2_ for 2 hours. As effector cells, PBMCs were used to kill the target cells, and the dead cells were then permeabilized to propidium iodide.

The PBMCs were first isolated and then washed twice with phosphate-buffered saline. For PBMC staining, trichromatic immunofluorescence analysis was performed on lymphocyte markers using an antibody immunofluorescence assay. The fluorescent dyes used for trichromatic immunofluorescence analysis were isothiocyanate, phycoerythrin, and allophycocyanin, which are anti-CD3 (Abcam, US, cat. no. ab237453), anti-CD16 (Abcam, US, cat. no. ab117117), and anti-CD56 (Abcam, US, cat. no. ab237383) antibodies labeled with fluorescein isothiocyanate. NK cells in peripheral blood were represented by CD3, CD56, and CD16 cells. PBMCs were used in this study when the cellular value and cytotoxicity of NK cells were greater than 14% and 15%, respectively. The frequencies of Th1 and Th2 cells were assessed using flow cytometry fluorescein isothiocyanate- (FITC-) labeled anti-IFN-*γ* (BioLegend, US, cat. no. 308703) and anti-IL-4-PE (BioLegend, US, cat. no. 355005) and were included in the analysis when the Th1/Th2 ratio was greater than 10.7% [16, 17].

### 2.6. Measurement of miRNA Expression Levels of T Cell-Related Markers

Total RNA was isolated from PBMCs using RNX-PLUS, and cDNA was synthesized using a reverse transcriptase kit. T cell-associated factors, T-bet, transcription factor GATA-binding protein 3 (GATA3), interferon regulatory factor 4 (IRF4), glucocorticoid-induced tumor necrosis factor (GITR), and tumor necrosis factor receptor superfamily 18 were evaluated using RT-PCR. The primers and reference genes are shown in Table 2. *β*-Actin was used as the reference gene, and the miRNA level was calculated as the ratio of the target miRNA genes to the reference gene. Relative expression was normalized and presented as the ratio to the relative gene in the control group.

### 2.7. Evaluation of miRNAs Associated with T Cell Subsets

The miRNAs associated with Treg (miR-106b-93-25, miR-146a, and miR-155) and Th17 (miR-326) cells in PBMCs were evaluated by RT-PCR on a LightCycler 2.0 RT-PCR system. The primers and the reference gene are shown in Table 3. RNU6B was used as the reference gene, and the miRNA level was calculated as the ratio of the target miRNA genes to the reference gene. Relative expression was normalized and presented as the ratio to the relative gene in the control group.

### 2.8. Assessment of T Cell Subpopulation Frequencies

Counting of Th17, Treg, exhausted T, and exhausted Treg cell was performed using flow cytometry. The monoclonal antibodies of surface and intracellular antigens used for counting Th17 cells were CD4-FITC (BioLegend, US, cat. no. 357405) and IL-17A-PE (BioLegend, US, cat. no. 506903), and CD4-FITC (BioLegend, US, cat. no. 357405), CD25-PE (BioLegend, US, cat. no. 985802), and CD127-PerCP-Cy5.5 (BioLegend, US, cat. no. 351321) were used for Treg cells; CD8-FITC (BioLegend, US, cat. no. 980908), PD-1-PerCP-Cy5.5 (BioLegend, US, cat. no. 329913), and Tim-3-PE (BioLegend, US, cat. no. 345006) for exhausted T cells; and CD4-FITC (BioLegend, US, cat. no. 357405), CD25-PE (BioLegend, US, cat. no. 985802), and PD1-PerCP-Cy5.5 (BioLegend, US, cat. no. 329913) for exhausted Treg cells [18].

### 2.9. Statistical Methods

SPSS 22.0 statistical software was used for statistical analysis. Data were expressed as mean ± standard deviation, and Student's *t*-test or chi-square test were used for between-group comparisons. Count data were expressed as ratios, and the data were processed by a chi-square test. *P* < 0.05 was considered a significant difference.

## 3. Results

### 3.1. Expression Levels of T Cell-Related Markers in the Two Groups

**Figure 1:**
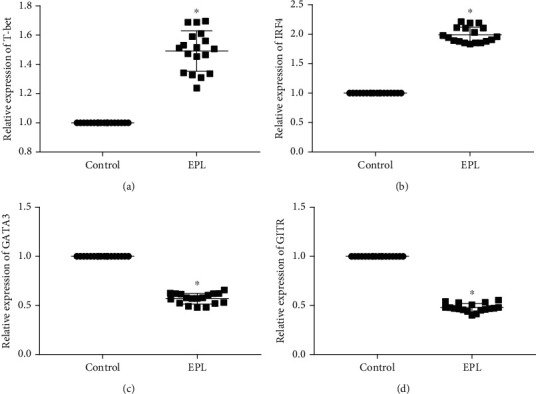
Expression of T cell-related markers in PBMCs in the EPL and control groups. (a) T-bet, (b) IRF 4, (c) GATA3, and (d) GITR. ^∗^ indicates *P* value was less than 0.05.

The expression levels of T cell subset-related genes in PBMCs in the two groups were compared. The results revealed significant differences in the mRNA expression levels of T-bet, GATA3, IRF4, and GITR (*P* < 0.05) (Figure 1). Compared with the control group, the mRNA expression levels of T-bet and IRF-4 in the PBMCs in the EPL group were significantly upregulated (*P* < 0.05), whereas those of GATA3 and GITR were significantly downregulated (*P* < 0.05).

### 3.2. Expression Levels of T Cell Subpopulation-Associated miRNAs in the Two Groups

**Figure 2:**
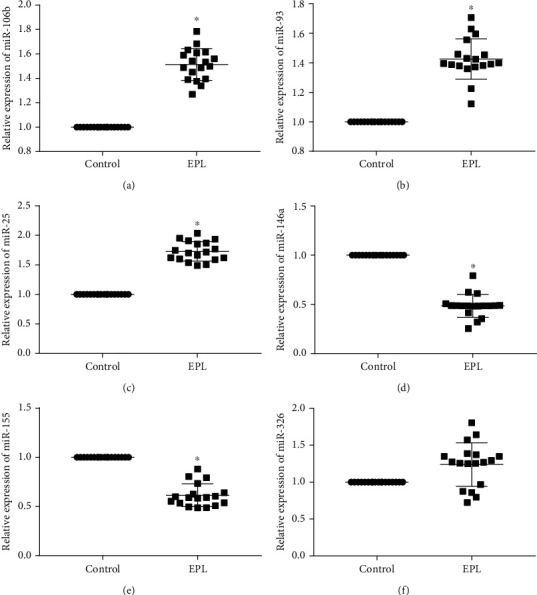
Expression of T cell subset-related miRNAs in the two groups. (a) miR-106b, (b) miR-93, (c) miR-25, (d) miR-146a, (e) miR-155, and (f) miR-326. ^∗^ indicates *P* value was less than 0.05.

The analysis of Treg cell-related miRNA revealed a significant increase in miR-25, miR-106b, and miR-93 expressions in the EPL group as compared with that in the control group (*P* < 0.05). In addition, the expression of miR-146a and miR-155 in PBMCs in the EPL group was significantly downregulated as compared with than that in the control group (*P* < 0.05). However, the expression of miR-326, which regulates the differentiation towards Th17 cells [19], in PBMCs in the EPL group was not significantly different from that in the control group (Figure 2).

### 3.3. Flow Cytometry Analysis of T Cell Subpopulation Frequencies in the Two Groups

**Figure 3:**
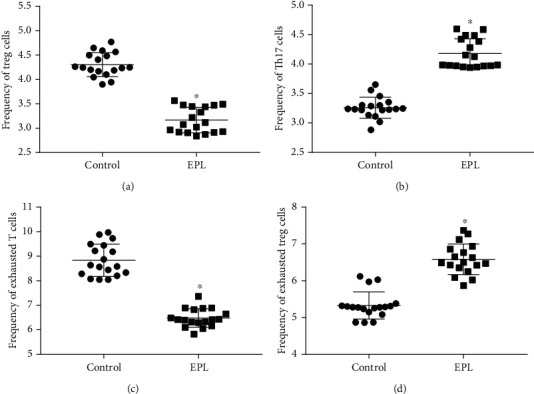
Frequency of T cell subsets in the two groups. (a) Treg cells, (b) Th17 cells, (c) exhausted T cells, and (d) exhausted Treg cells. ^∗^ indicates *P* value was less than 0.05.

The results of flow cytometry showed that the frequency of peripheral blood Treg cells and exhausted T cells in the EPL group was significantly lower than that in the control group (*P* < 0.05). And the frequency of Th17 cells and exhausted Treg cells in peripheral blood in the EPL group was significantly increased as compared with than that in the control group (*P* < 0.05) (Figure 3).

## 4. Discussion

The incidence of EPL in women of normal childbearing age is approximately 1–3%, with the causes primarily linked to chromosomal, endocrine, anatomical, infectious, and immunological abnormalities [6, 7]. However, in most cases, the cause of EPL remains unexplained. In recent years, some studies have investigated various aspects of recurrent EPL, including genes associated with folate metabolism [20], oxidative stress levels, sperm DNA damage [6], congenital uterine malformations in pregnant women, and acquired coagulation dysfunction, all of which have made breakthroughs.

Recurrent EPL may be related to T cell subsets and their associated miRNAs. To shed light on this issue, the present study investigated the expression of T cell subsets and their associated miRNAs in patients with recurrent EPL. Previous studies reported that CD4^+^, CD25^+^, and Treg cells were downregulated in peripheral lymphocytes and metaphase lymphocytes of women who had a miscarriage [21, 22]. Research also showed that the frequency of Th17 cells in peripheral blood lymphocytes and metaphase lymphocytes was increased in women with recurrent miscarriages as compared with that in women who had normal pregnancies [23]. In addition, a negative correlation between Th17 and Treg lymphocytes was found in the peripheral blood of these patients [23]. The ratio of Th17/Treg is increased in women with EPL, signifying a proinflammatory response, which may be accompanied by a decrease in the regulatory response, potentially leading to the development of RPL [24]. In this study, the expression frequency of Th17 in the EPL group was higher than that in the control group, whereas the expression of Treg cells showed a decreasing trend compared with the control group, which is consistent with the previous research [24]. As reported previously, miR-155 induces the differentiation of Treg and Th17, as well as IL-17A secretion by Th17 cells, whereas it has no significant effect on the secretion of Treg-related cytokines [25]. In addition, miR-146a has been shown to be essential for the suppressive function of Treg cells, with regulation of the Th1 response controlled by miR-146a expression in Treg cells [26]. Furthermore, miR-146a may be able to inhibit the transformation of Treg cells to Th1-like cells. In PBMCs cocultured with colorectal cancer cells, miR-146a increased the number of Treg cells and associated suppressor cytokines, such as transforming growth factor-*β* (TGF-*β*) and IL-10 [26, 27]. According to our results, the expression of both miR-155 and miR-146a seems to be reduced in patients with recurrent EPL, which may be related to the reduced number of Treg cells in the patients. Studies have shown that the number of Treg cells decreased in both mouse pregnancy loss model and patients with unexplained recurrent pregnancy loss [28, 29].

The miR-106b-93-25 cluster plays a crucial role in the regulation of the TGF-*β* signaling pathway, and it is an essential cytokine in the induction of Foxp3 production by Treg cells. Foxp3 is a transcription factor which has been found to play a critical role in the control of inflammation [30]. Increased expression of miR-106b-25 may disrupt the TGF-*β* signaling pathway, which plays an important role in maintaining the function and inducing the generation of Treg cells. Our results revealed a decrease in the number of Treg cells and an increase in the transcript level of the miR-106b-93-25 cluster in the peripheral blood of patients in the EPL group. We speculate that there is a definite link between them and that miR-106b-93-25 controls the mechanobiology of Treg cells in women with EPL [31–33].

The results of this study revealed an uncontrolled inflammatory microenvironment in the EPL group, as demonstrated by the increased frequency of Th17 and exhausted Treg cells and increased mRNA expression of T-bet and IRF-4 in peripheral blood. In this study, failure to suppress inflammation was followed by a decrease in the frequencies of Treg and exhausted T cells, as well as a decrease in mRNA expression of GATA3 and GITR. The negative expression of above factors adversely affect the gestational process and may lead to miscarriages. Thus, immunological parameters and their associated epigenetic factors, such as the frequency of Th17 and exhausted Treg cells and expression of T cell-associated factors, may be used as prognostic biomarkers to prevent immune miscarriage in women at high risk.

In conclusion, during pregnancy, regulatory and inhibitory mechanisms control the immune system, possibly via miRNAs. Dysregulation of the immune system may lead to rejection of the embryo and EPL. In this study, we compared T cell-related markers in peripheral blood of patients with recurrent EPL and women with normal pregnancies. The results showed that the expression of T-bet and IRF4 was significantly upregulated, whereas the expression of GATA3 and GITR was significantly downregulated. Among the T cell subpopulation of Treg cell-associated miRNAs, in the EPL group, the expression of miR-106b, miR-93, and miR-25 was upregulated, whereas the expression of miR-146a and miR-155 was downregulated. Compared with the control group, the frequencies of Treg and exhausted T cells were decreased, whereas those of Th17 and exhausted Treg cells were increased in the EPL group. There were significant differences in the expression and frequency of T cell subsets and their related miRNAs in the two groups. Therefore, immune cells and their associated miRNA can be used as prognostic biomarkers for the treatment of human reproductive disorders in clinical studies.

## Figures and Tables

**Table 1:** Comparison of general data in the two groups.

	EPL group (*n* = 50)	Control group (*n* = 50)	*t*/*χ*^2^	*P*
Age	27.28 ± 3.10	28.35 ± 3.56	1.10	0.75
Body mass index (kg/m^2^)	19.87 ± 2.38	20.0 ± 2.58	2.08	0.54
Gravidity			4.04	0.23
2	13 (26)	23 (46)		
3	26 (52)	20 (40)		
≥4	11 (22)	7 (14)		
Parity			3.56	0.28
0	7 (14)	9 (18)		
1	22 (44)	26 (52)		
≥2	21 (42)	15 (30)		

**Table 2:** Primer sequences of target and reference genes.

Genes	Primer	Sequence (5′→3′)
TBX-21 (T-bet)	Forward	GGTGAACGACGGAGAGC
	Reverse	TCGGCATTCTGGTAGGC
GATA-3	Forward	GCCTCAGCCACTCCTAC
	Reverse	CCTGACCGAGTTTCCGTAG
IRF-4	Forward	CGTTCATTGCTCTCCAGTCAC
	Reverse	GCCTTCACGCACCATTCAG
TNFRSF18 (GITR)	Forward	TGGGTCGGGATTCTCAGGTC
	Reverse	TTTCAAGAGCCCACAGCCAG
*β*-Actin	Forward	GCATGGGTCAGAAGGATTCCT
	Reverse	TCGTCCCAGTTGGTGACG

**Table 3:** Primer sequences of the miRNAs and reference gene.

Genes	Primer	Sequence (5′→3′)
miR-106b	Forward	GGGGCTAAAGTGCTGACAGT
	Reverse	GGAGCAGCAAGTACCCACAG
miR-93	Forward	CTCCAAAGTGCTGTTCGTGC
	Reverse	GGGGCTCGGGAAGTGCTA
miR-25	Forward	GTGTTGAGAGGCGGAGACTT
	Reverse	TGTCAGACCGAGACAAGTGC
miR-146a	Forward	ACTGAATTCCATGGGTTGTGTC
	Reverse	TGACAGAGATATCCCAGCTGAAG
miR-155	Forward	TGCGCTTAATGCTAATTGTGATA
	Reverse	CCAGTGCAGGGTCCGAGGTATT
miR-326	Forward	CATCTGTCTGTTGGGCTGGA
	Reverse	TGGAGGAAGGGCCCAGAG
RNU6B	Forward	CTCGCTTCGGCAGCACA
	Reverse	AAGGCTTCACGAATTTGCGT

## Data Availability

The data of this manuscript are available from the corresponding author upon request.

## References

[1] Li L., Liu J., Qin S., Li R. (2018). The association of polymorphisms in promoter region of MMP2 and MMP9 with recurrent spontaneous abortion risk in Chinese population. *Medicine*.

[2] Na E. D., Jung I., Choi D. H. (2018). The risk factors of miscarriage and obstetrical outcomes of intrauterine normal pregnancy following heterotopic pregnancy management. *Medicine*.

[3] Quenby S., Gallos I. D., Dhillon-Smith R. K. (2021). Miscarriage matters: the epidemiological, physical, psychological, and economic costs of early pregnancy loss. *Lancet (London, England)*.

[4] Kim D. J., Lee S. K., Kim J. Y. (2014). Intravenous immunoglobulin G modulates peripheral blood Th17 and Foxp3(+) regulatory T cells in pregnant women with recurrent pregnancy loss. *American journal of reproductive immunology (New York, NY: 1989)*.

[5] Xie M., Li Y., Meng Y. Z. (2022). Uterine natural killer cells: a rising star in human pregnancy regulation. *Frontiers in Immunology*.

[6] Zidi-Jrah I., Hajlaoui A., Mougou-Zerelli S. (2016). Relationship between sperm aneuploidy, sperm DNA integrity, chromatin packaging, traditional semen parameters, and recurrent pregnancy loss. *Fertility and Sterility*.

[7] Sapra K. J., Joseph K. S., Galea S., Bates L. M., Louis G. M., Ananth C. V. (2017). Signs and symptoms of early pregnancy loss. *Reproductive sciences (Thousand Oaks, Calif)*.

[8] Muyayalo K. P., Li Z. H., Mor G., Liao A. H. (2018). Modulatory effect of intravenous immunoglobulin on Th17/Treg cell balance in women with unexplained recurrent spontaneous abortion. *American journal of reproductive immunology (New York, NY: 1989)*.

[9] Hadfield K. A., McCracken S. A., Ashton A. W., Nguyen T. G., Morris J. M. (2011). Regulated suppression of NF-*κ*B throughout pregnancy maintains a favourable cytokine environment necessary for pregnancy success. *Journal of Reproductive Immunology*.

[10] McCracken S. A., Hadfield K., Rahimi Z., Gallery E. D., Morris J. M. (2007). NF-*κ*B-regulated suppression of T-bet in T cells represses Th1 immune responses in pregnancy. *European Journal of Immunology*.

[11] Qin W., Tang Y., Yang N., Wei X., Wu J. (2016). Potential role of circulating microRNAs as a biomarker for unexplained recurrent spontaneous abortion. *Fertility And Sterility*.

[12] Cui S., Zhang J., Li J. (2021). Circulating microRNAs from serum exosomes as potential biomarkers in patients with spontaneous abortion. *American Journal of Translational Research*.

[13] Liu H. N., Tang X. M., Wang X. Q. (2020). MiR-93 inhibits trophoblast cell proliferation and promotes cell apoptosis by targeting BCL2L2 in recurrent spontaneous abortion. *Reproductive sciences (Thousand Oaks, Calif)*.

[14] Zhang Q., Tian P., Xu H. (2021). MicroRNA-155-5p regulates survival of human decidua stromal cells through NF-*κ*B in recurrent miscarriage. *Reproductive Biology*.

[15] American College of Obstetricians and Gynecologists (2015). The American College of Obstetricians and Gynecologists practice bulletin no. 150. Early pregnancy loss. *Obstetrics & Gynecology*.

[16] Abdolmohammadi Vahid S., Ghaebi M., Ahmadi M. (2019). Altered T-cell subpopulations in recurrent pregnancy loss patients with cellular immune abnormalities. *Journal of Cellular Physiology*.

[17] Ahmadi M., Abdolmohammadi-Vahid S., Ghaebi M. (2017). Effect of intravenous immunoglobulin on Th1 and Th2 lymphocytes and improvement of pregnancy outcome in recurrent pregnancy loss (RPL). *Biomedicine & Pharmacotherapy = Biomedecine & Pharmacotherapie*.

[18] Ahmadi M., Abdolmohammadi-Vahid S., Ghaebi M. (2017). Regulatory T cells improve pregnancy rate in RIF patients after additional IVIG treatment. *Systems Biology in Reproductive Medicine*.

[19] Vega-Cárdenas M., Uresti-Rivera E. E., Cortés-García J. D. (2019). Increased levels of adipose tissue-resident Th17 cells in obesity associated with miR-326. *Immunology Letters*.

[20] Nowak I., Bylińska A., Wilczyńska K. (2017). The methylenetetrahydrofolate reductase c.c.677 C>T and c.c.1298 A>C polymorphisms in reproductive failures: experience from an RSA and RIF study on a Polish population. *PLoS One*.

[21] Jin L. P., Chen Q. Y., Zhang T., Guo P. F., Li D. J. (2009). The CD4+CD25 bright regulatory T cells and CTLA-4 expression in peripheral and decidual lymphocytes are down-regulated in human miscarriage. *Clinical Immunology*.

[22] Winger E. E., Reed J. L. (2011). Low circulating CD4(+) CD25(+) Foxp3(+) T regulatory cell levels predict miscarriage risk in newly pregnant women with a history of failure. *American journal of reproductive immunology (New York, NY: 1989)*.

[23] Wang W. J., Hao C. F., Yi L. (2010). Increased prevalence of T helper 17 (Th17) cells in peripheral blood and decidua in unexplained recurrent spontaneous abortion patients. *Journal of Reproductive Immunology*.

[24] Lee S. K., Kim J. Y., Hur S. E. (2011). An imbalance in interleukin-17-producing T and Foxp3^+^ regulatory T cells in women with idiopathic recurrent pregnancy loss. *Human Reproduction (Oxford, England)*.

[25] Yao R., Ma Y. L., Liang W. (2012). MicroRNA-155 modulates Treg and Th17 cells differentiation and Th17 cell function by targeting SOCS1. *PLoS One*.

[26] Lu L. F., Boldin M. P., Chaudhry A. (2010). Function of miR-146a in controlling Treg cell-mediated regulation of Th1 responses. *Cell*.

[27] Khorrami S., Zavaran Hosseini A., Mowla S. J., Soleimani M., Rakhshani N., Malekzadeh R. (2017). MicroRNA-146a induces immune suppression and drug-resistant colorectal cancer cells. *Tumour biology: the journal of the International Society for Oncodevelopmental Biology and Medicine*.

[28] Kedzierska A. E., Lorek D., Slawek A., Grabowski T., Chelmonska-Soyta A. (2021). CD91 derived treg epitope modulates regulatory T lymphocyte response, regulates expression of costimulatory molecules on antigen-presenting cells, and rescues pregnancy in mouse pregnancy loss model. *International journal of molecular sciences*.

[29] Zhang Z., Long S., Huang Z., Tan J., Wu Q., Huang O. (2020). Regulatory effect of daphnetin on the balance of Th17 and Treg cells in the peripheral blood mononuclear cells from patients with unexplained recurrent pregnancy loss. *Central-European Journal Of Immunology*.

[30] Arpaia N., Campbell C., Fan X. (2013). Metabolites produced by commensal bacteria promote peripheral regulatory T-cell generation. *Nature*.

[31] Petrocca F., Vecchione A., Croce C. M. (2008). Emerging role of miR-106b-25/miR-17-92 clusters in the control of transforming growth factor beta signaling. *Cancer Research*.

[32] Fantini M. C., Becker C., Monteleone G., Pallone F., Galle P. R., Neurath M. F. (2004). Cutting edge: TGF-beta induces a regulatory phenotype in CD4+CD25- T cells through Foxp3 induction and down-regulation of Smad7. *Journal of immunology (Baltimore, Md: 1950)*.

[33] De Santis G., Ferracin M., Biondani A. (2010). Altered miRNA expression in T regulatory cells in course of multiple sclerosis. *Journal of Neuroimmunology*.

